# Role of Autoantibodies in the Diagnosis of Connective-Tissue Disease ILD (CTD-ILD) and Interstitial Pneumonia with Autoimmune Features (IPAF)

**DOI:** 10.3390/jcm6050051

**Published:** 2017-05-04

**Authors:** Adelle S. Jee, Stephen Adelstein, Jane Bleasel, Gregory J. Keir, MaiAnh Nguyen, Joanne Sahhar, Peter Youssef, Tamera J. Corte

**Affiliations:** 1 Department of Respiratory and Sleep Medicine, Royal Prince Alfred Hospital, Sydney, NSW 2050, Australia; adelle.jee@sswahs.nsw.gov.au; 2 Sydney Medical School, University of Sydney, Sydney, NSW 2006, Australia; stephen.adelstein@sydney.edu.au (S.A.); jane.bleasel@sydney.edu.au (J.B.); maianh.nguyen@sswahs.nsw.gov.au (M.N.); pyoussef@med.usyd.edu.au (P.Y.); 3 Immunopathology Laboratory, Southwest Sydney Pathology Service, Sydney, NSW 2050, Australia; 4 Department of Clinical Immunology and Allergy, Royal Prince Alfred Hospital, Sydney, NSW 2050, Australia; 5 Department of Rheumatology, Royal Prince Alfred Hospital, Sydney, NSW 2050, Australia; 6 Department of Respiratory, Princess Alexandra Hospital, Woolloongabba, QLD 4102, Australia; gregory_keir@health.qld.gov.au; 7 Department of Rheumatology, Monash Health, Clayton, VIC 3168, Australia; josahhar@bigpond.com; 8 Department Medicine, Monash University, Clayton, VIC 3168, Australia

**Keywords:** interstitial lung disease, connective tissue disease, autoantibodies, interstitial pneumonia with autoimmune features, diagnosis

## Abstract

The diagnosis of interstitial lung disease (ILD) requires meticulous evaluation for an underlying connective tissue disease (CTD), with major implications for prognosis and management. CTD associated ILD (CTD-ILD) occurs most commonly in the context of an established CTD, but can be the first and/or only manifestation of an occult CTD or occur in patients who have features suggestive of an autoimmune process, but not meeting diagnostic criteria for a defined CTD—recently defined as “interstitial pneumonia with autoimmune features” (IPAF). The detection of specific autoantibodies serves a critical role in the diagnosis of CTD-ILD, but there remains a lack of data to guide clinical practice including which autoantibodies should be tested on initial assessment and when or in whom serial testing should be performed. The implications of detecting autoantibodies in patients with IPAF on disease behaviour and management remain unknown. The evaluation of CTD-ILD is challenging due to the heterogeneity of presentations and types of CTD and ILD that may be encountered, and thus it is imperative that immunologic tests are interpreted in conjunction with a detailed rheumatologic history and examination and multidisciplinary collaboration between respiratory physicians, rheumatologists, immunologists, radiologists and pathologists.

## 1. Background

Interstitial lung diseases (ILDs) encompass chronic lung disorders characterised by damage to lung tissue by inflammation and/or fibrosis. An accurate ILD diagnosis requires the meticulous evaluation for any potential aetiology, including environmental exposures, medications, and especially connective tissue diseases (CTDs), with major implications for management and prognosis [1,2,3,4]. CTDs are a group of autoimmune disorders, including rheumatoid arthritis (RA), systemic lupus erythematosus (SLE), idiopathic inflammatory myopathies including polymyositis/dermatomyositis (IIM; PM/DM), Sjögren’s syndrome (SS), systemic sclerosis (SSc) and mixed connective tissue disease (MCTD) [5]. ILD can be associated with all CTDs and may present in one of three settings: (a) ILD in a patient with an established CTD; (b) ILD as the first and/or only manifestation of the CTD; (c) ILD with some autoimmune features, but not fulfilling criteria for a defined CTD—recently defined as “interstitial pneumonia with autoimmune features” (IPAF) in an European Respiratory Society/American Thoracic Society statement to standardise terminology for future research [5,6,7,8,9,10].

The reported frequency of ILD associated with each CTD is highly variable, influenced by disease-specific and demographic factors, complicated by differences in disease definitions and intensity of screening practices worldwide. The diagnosis of CTD associated ILD (CTD-ILD) is challenging and requires careful evaluation of clinical, physiological, radiological, histopathological and immunological markers of disease. For these reasons, a multidisciplinary approach has become the gold standard in ILD diagnosis, involving close communication between pulmonologists, rheumatologists, immunologists, radiologists and pathologists in a dynamic process that may require repeating as a patient’s disease evolves [11].

This review summarises the literature regarding the utility of CTD-associated autoantibodies in the diagnosis of CTD-ILD, potential implications for ILD patients with positive autoantibodies but without full diagnostic features of a CTD, and current gaps in the available evidence. Although autoantibodies have demonstrable utility in the diagnosis and management of patients with CTD-ILD, there remains a lack of data to guide clinical practice.

## 2. Diagnosis of CTD-ILD

### 2.1. Clinical Features of CTD-ILD

In general, patients with CTD-ILD are more likely to be younger, female and non-smokers compared with those with idiopathic interstitial pneumonia (IIP) [9]. Extrathoracic manifestations depend on the underlying CTD, but highly suggestive features include Raynaud’s phenomenon, inflammatory arthritis, pleuritis and/or pericarditis, sicca symptoms (dry eyes and dry mouth), rash and oesophageal dysmotility.

Nailfold capillaroscopy (NFC) is a non-invasive technique in which a magnifying lens is used to assess the nail bed microcirculation [12]. NFC is included in current European League Against Rheumatism (EULAR) classification criteria for systemic sclerosis (SSc) diagnosis and characteristic findings include decreased capillary density, neo-vascularisation and micro-haemorrhages [13,14]. There is increasing evidence that qualitative and quantitative assessment of nailfold changes identifies individuals with “early” SSc and is predictive of risk of disease progression and future complications, such as digital trophic lesions [15]. Non-specific NFC abnormalities have been demonstrated in CTDs other than SSc and IIP (predominantly idiopathic pulmonary fibrosis; IPF), and the role of NFC in differentiating CTD-ILD from IIP remains to be determined [12,16].

### 2.2. Radiological Features of CTD-ILD

All radiologic patterns of interstitial pneumonia can be observed in CTD-ILD, with the frequency of each pattern depending on the specific underlying CTD [17]. Overall, the most common radiologic pattern is non-specific interstitial pneumonia (NSIP), which can be found in all CTDs, but particularly SSc and polymyositis/dermatomyositis (PM/DM) [18,19]. NSIP is characterised by temporally homogeneous ground glass opacities (GGO), mild reticulation and bronchiectasis, in a predominantly peri-bronchovascular distribution [20]. Distinctive sparing of the sub-pleural lung zone may be seen, and honeycombing may occur in advanced stages but is rare [18,19]. NSIP associated with CTD is more common than idiopathic NSIP, thus a patient presenting with NSIP should be thoroughly investigated and monitored for an underlying or evolving CTD, even in the absence of extrathoracic manifestations of autoimmune disease [21].

Usual interstitial pneumonia (UIP) is the second most common radiologic pattern seen in CTD-ILD, and the most common pattern identified in rheumatoid arthritis (RA) [17]. The UIP pattern seen in CTD-ILD is indistinguishable from that of IPF, and characterised by honeycombing, reticulation and traction bronchiectasis, in a predominantly sub-pleural, basal distribution [17,22]. Ground glass opacities are uncommon and need to be distinguished from early or fine fibrosis [20].

Other radiologic patterns that may be identified in CTD-ILD include organising pneumonia (OP), most commonly associated with PM/DM, characterised by bilateral patchy consolidation or GGO in a peripheral, peri-bronchovascular or band-like distribution, and may manifest as the “reversed halo” sign; a mixed NSIP with OP overlap pattern, associated with idiopathic inflammatory myopathies and the anti-synthetase syndrome; and less commonly, lymphoid interstitial pneumonia (LIP), most closely associated with Sjogren’s syndrome and demonstrating GGO with scattered thin-walled cysts in a perivascular distribution; and diffuse alveolar damage (DAD) during acute exacerbations [20,22].

Other radiological features that may suggest an underlying CTD include multi-compartment abnormalities such as oesophageal dilatation and pleural and/or pericardial involvement. In patients with an established CTD on immunosuppressive treatment, confounding factors that may mimic ILD and require exclusion include drug toxicity, infection and CTD-associated malignancy [20].

### 2.3. Histopathological Features of CTD-ILD

In the presence of CTD, surgical lung biopsy is seldom required to confirm ILD as clinical features and imaging are often sufficient to confidently distinguish CTD-ILD from IIP. In addition, the underlying histopathological pattern has less impact on management and prognosis compared with IIP. Unlike IIP where a pattern of UIP is associated with significantly worse survival compared with fibrotic NSIP, distinguishing UIP from NSIP in the context of SSc-ILD does not appear to delineate outcomes or survival [23,24]. RA-ILD represents a possible exception, with some evidence suggesting worse survival with in RA-UIP compared with RA-NSIP [2,25]. Data regarding outcomes in CTD-ILD with histopathological patterns other than UIP or NSIP is limited.

In cases where surgical lung biopsy is performed, NSIP is the most common pattern identified, except in RA where UIP predominates. Other patterns observed include organising pneumonia (OP), desquamative interstitial pneumonia (DIP), lymphocytic interstitial pneumonia (LIP), diffuse alveolar damage (DAD) and overlap patterns [26,27]. Other features that may prompt closer investigation for an underlying CTD include additional involvement of airways, vasculature or pleura, and evidence of inflammation, germinal centre formation, increased perivascular collagen, follicular bronchiolitis, lymphoplasmacytic inflammation, eosinophilic infiltration or pleuritis [26,28,29,30].

The prevalence of ILD and associated radiological–histological pattern for each of the major CTDs is summarised in Table 1.

### 2.4. Role of Autoantibodies in the Diagnosis of CTD-ILD

The detection of specific autoantibodies serves a critical role in the diagnosis of CTD-ILD and also carries value predicting outcomes and guiding management. The major autoantibodies and their associated CTDs are shown in Table 2.

The detection of specific autoantibodies in an individual with ILD may point towards or represent a “forme fruste” presentation of an underlying autoimmune process, with the potential to alter diagnosis from idiopathic ILD to CTD-ILD. The detection of autoantibodies may assist the diagnosis of an unrecognised CTD in specialist ILD clinics with a reported frequency of 4% to 19% [7,37,38,39]. In established CTD-ILD, autoantibodies can also allow more precise classification between the various CTDs presenting with similar clinical, radiological and/or histopathological features [39].

Distinguishing CTD-ILD from IIP is critical and has major implications for prognosis and management. The long-term prognosis of CTD-ILD is generally less severe than that of the most common IIP, idiopathic pulmonary fibrosis (IPF). Better understanding of underlying disease mechanisms has also resulted in increasingly targeted therapies. Approved for clinical use in 2014, novel anti-fibrotic therapies nintedanib and pirfenidone have shown the ability to slow disease progression in IPF and are now available in many countries [40,41]. Conversely, immunosuppression forms the core treatment for CTD-ILD (including corticosteroids, other immunosuppressive agents such as mycophenolate mofetil and newer biological agents such as rituximab), and have shown harm in IPF [42]. Furthermore, identifying CTD-ILD has consequences for the screening of other systemic manifestations and complications (for example, pulmonary arterial hypertension in systemic sclerosis) and the choice of disease modifying agent used for the primary CTD [21].

The critical role of autoantibodies in the diagnosis and management of patients with ILD is emphasised by the 2011 American Thoracic Society/European Respiratory Society/Japanese Respiratory Society and Latin American Thoracic Association (ATS/ERS/JRS/ALAT) guidelines for the diagnosis of ILD, which recommend testing for antinuclear antibody (ANA), anti-cyclic citrullinated peptide (anti-CCP) and rheumatoid factor (RF) in all patients with suspected ILD, even without overt features of a CTD [1]. More specific tests, including extractable nuclear antigen (ENA) antibodies (e.g., anti-Scl70, SSA/Ro, SSB/La, RNP and Sm autoantibodies), and selected myositis antibodies (anti-Jo1, PL-7 and PL-12), are recommended only in selected cases [1].

However, the diagnostic value and cost-effectiveness of this approach has not been formally evaluated and there remains no universal consensus which autoantibodies should be tested on initial screening and subsequent testing. The above approach may fail to detect an underlying CTD in patients who present with pulmonary signs and symptoms as their first manifestation of CTD-ILD and have absent or subtle extrathoracic features. This importantly includes the inflammatory myopathies, especially “anti-synthetase syndrome” and “clinically amyopathic dermatomyositis”, which carry a substantially increased risk of ILD and high mortality and are identified by the presence of anti-synthetase or anti-CADM140/MDA5 antibodies and may be present in patients with apparent “idiopathic” ILD without ANA or anti-Jo1 antibodies on initial serological screening [35,43].

There is also increasing recognition and interest in ILD patients in whom CTD-associated antibodies are detected, but who do not fulfil criteria for a CTD—some of whom would now be identifiable as IPAF. Although largely still a research classification, the serological domain of the IPAF criteria requires evaluation for a broader array of autoantibodies (including an extended ENA and myositis panel), recognising the low specificity of a low ANA titre and RF level [10]. The authors recognise that the proposed criteria and included specific autoantibodies will require validation and revision with further study, and in the absence of any other clinical or morphological features of a CTD they would remain classified as idiopathic ILD.

The ATS/ERS/JRS/ALAT diagnostic guidelines also recommend serial serological testing to identify patients who seroconvert and become autoantibody positive during follow-up and may require a revision in diagnosis (e.g., from idiopathic ILD to CTD-ILD) [1]. A recent study of 1044 Chinese CTD-ILD patients demonstrated seroconversion in 51.2% of patients at follow-up, 32% of which were initially given an alternative ILD diagnosis most likely due to the absence of autoantibodies and overt rheumatologic features at first assessment [44]. However, there remains a paucity of data to guide when or in whom serial testing should be performed. 

**Figure 1:**
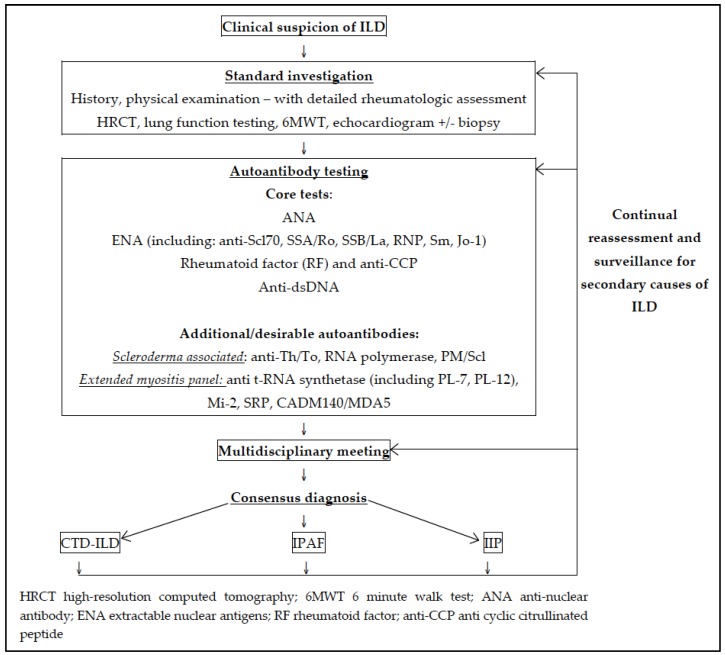
Suggested algorithm for assessment of interstitial lung disease and autoantibody testing. HRCT: high-resolution computed tomography; 6MWT: 6-minute walk test; ANA: anti-nuclear antibody; ENA: extractable nuclear antigens; RF: rheumatoid factor; anti-CCP: anti cyclic citrullinated peptide.

Thus, despite the crucial role autoantibodies demonstrate in the diagnosis and management of patients with ILD, there remains a lack of data to guide clinical practice. Figure 1 illustrates a suggested algorithm for the assessment of ILD and how autoantibody testing may fit in this schema based on the current literature.

## 3. CTD Associated Autoantibodies

### 3.1. Antinuclear Antibodies (ANA) and Antibodies Associated with Systemic-Sclerosis

ILD occurs more often in SSc than any other CTD and is the leading cause of mortality in SSc [45]. Antinuclear antibodies (ANAs) are autoantibodies directed against antigens in the cell nucleus, and recommended for all patients undergoing assessment of ILD [46]. Indirect immunofluorescence is the recommended screening method for ANA, allowing evaluation of both antibody titre and staining pattern [47]. A low titre ANA (1:40) can be found in 25–30% of healthy individuals and occurs with greater frequency in females and older individuals [48,49]. Laboratory thresholds for a positive test vary, but as a guide an ANA titre greater than or equal to 1:160 has greater utility, being detected in only 5% of healthy individuals [49]. However, a negative test at a titre of 1:40 has a high negative predictive value in excluding a CTD.

Up to 56% of patients with a new diagnosis of ILD have a positive ANA at baseline [7]. The utility of ANA in detecting unrecognised or confirming CTD diagnosis is greatest in young patients and individuals with SSc and SLE. In the right clinical context, ANA is also useful in Sjögren’s syndrome and PM/DM [7,49]. However, some CTDs can be ANA-negative (e.g., anti-synthetase syndrome) and false negatives do occur. Thus, additional immunologic tests such as ENA and myositis specific antibodies and further rheumatologic evaluation should be pursued if clinically suspected.

In SSc, 75–95% of patients demonstrate ANA, with an overall diagnostic sensitivity of 85% and specificity of 54% [49,50]. There are at least seven SSc-specific antibodies, each varying in their frequency and clinical significance (including their association with SSc associated ILD; SSc-ILD), influenced by laboratory technique, demographic and environmental factors [50,51]. The three ANAs that are most frequently associated with SSc include anti-centromere antibodies (ACA), anti-topoisomerase I antibodies (ATA; also known as anti-Scl70) and anti-RNA polymerase (anti-RNA pol) antibodies [52]. Occurring in over 50% of patients with SSc, they are highly specific and generally present exclusive of each other [52,53].

#### 3.1.1. Anti-topoisomerase I antibodies (ATA; anti-Scl70)

Anti-topoisomerase I antibodies (ATA), also known as anti-Scl70, occur in SSc with a frequency of approximately 28% (range 9.4% to 42%) [53]. ATA is highly specific for SSc (90–100%), and is associated with diffuse cutaneous SSc (although still observed in limited forms), poor prognosis, and higher risk of pulmonary fibrosis [54,55,56]. Sensitivity and specificity for predicting radiographic ILD in SSc is approximately 45% and 81% respectively [57]. Other disease associations include digital ulcers and cardiac, muscle and joint involvement [51,55,58,59]. Within ATA-positive SSc populations, African American patients have more frequent and more severe pulmonary fibrosis, with lower survival rates compared with Caucasians [60]. There is currently little evidence for serial measurement of ATA, and the predictive utility of ATA remains unclear with conflicting reports on whether titres correlate with fibrosis extent on high-resolution computed tomography (HRCT) and/or degree of impairment by pulmonary function measures [32,57,61].

#### 3.1.2. Anti-Centromere Antibodies (ACA)

Anti-centromere antibodies (ACA) are one of the most frequently observed autoantibodies in SSc with a reported prevalence of 20–40% and specificity of 97% [53,55,57]. The majority of ACA-positive patients have limited SSc, and although there is an association with intrinsic pulmonary hypertension, several studies show relative protection from SSc-ILD [51,55,58,59,62].

#### 3.1.3. Anti-RNA Polymerase (RNA pol) Antibodies

Autoantibodies to the three mammalian RNA polymerases (RNA pol-I, II, III) are highly specific for SSc (98–100%), with an approximate prevalence of 20% in SSc (range 5–22%) [53,54]. Anti-RNA pol-II is less specific and detected in SLE and overlap syndromes [63]. The presence of anti-RNA pol-I and III has been associated with diffuse cutaneous involvement and higher risk of renal crisis, synovitis, myositis, joint contractures, and malignancy, but no specific association with ILD has been described [64,65,66].

#### 3.1.4. Other SSc-Associated Autoantibodies

##### Anti Th/To Antibodies

Anti-Th/To antibodies are 99% specific for SSc but relatively rare (prevalence 1–7%) [49,52,54,55]. Similar to ACA, anti-Th/To antibodies are associated with limited-SSc. However, they appear to occur exclusive to ACA and unlike ACA, are associated with reduced survival, increased risk of SSc-ILD and intrinsic pulmonary hypertension [52,67]. Limiting their clinical use is the current requirement for specialised testing and low antibody prevalence.

##### Anti-PM/Scl Antibodies

The PM/Scl antigen includes 16 target proteins, of which the 75 and 100 kDa proteins (PM/Scl-75 and PM/Scl-100) are the most frequently recognised [55]. Anti-PM/Scl antibodies are found in 4–11% of SSc patients, with anti-PM/Scl-75 the most common [68]. Detection of these antibodies occurs largely in European and US populations, with almost complete absence in Japanese cohorts [52]. Anti-PM/Scl antibodies are largely observed in PM/DM-SSc overlap syndromes, SLE and Sjögren’s syndrome [68]. Although rare, these antibodies are associated with increased risk of ILD and digital ulceration, whilst protecting against pulmonary hypertension [50,69,70].

##### Antibodies to Small Nuclear Ribonucleoprotein (Anti-U3, anti-U1 RNP)

Serum autoantibodies to small nuclear ribonucleoproteins (RNP) are relatively rare, but of these, anti-U1RNP and anti-U3RNP antibodies are the most frequently observed [71]. Anti-U1RNP antibodies are largely associated with mixed connective tissue disease (MCTD), but also seen in 2–14% of SSc-overlap syndromes [52,55]. One observational study reported a higher prevalence of ILD in anti-U1RNP positive SSc patients [58]. Anti-U3RNP antibodies are specific for SSc with a prevalence of 4–10% [55]. Several small studies report increased frequency of anti-U3-RNP in African American SSc patients and increased risk of intrinsic pulmonary hypertension, although association with ILD is conflicting [60,72,73,74].

##### Anti-Histone Antibodies

Anti-histone antibodies have been observed in SSc as well as drug induced lupus, SLE and RA [75]. Whilst a trend towards greater impairment of pulmonary function and frequency of pulmonary fibrosis has been demonstrated in small SSc cohorts, this is not confirmed by all studies [59,75,76,77].

##### Other

Other antibodies that have been observed in SSc with conflicting or minimal data regarding association with SSc-ILD include antibodies to platelet derived growth factor receptor (PDGFR), endothelial cells, activating transcription factor-2 (ATF-2), peroxiredoxin I (Prx I) and B23/nucleophosmin/numatrin [55,78,79,80,81].

### 3.2. Rheumatoid Factor (RF) and Anti-Citrullinated Cyclic Peptide Antibodies (anti-CCP)

Rheumatoid arthritis (RA) is associated with a variety of pulmonary manifestations including ILD (RA-ILD), parenchymal nodules, inflammatory pleural disease and pulmonary vascular disease (vasculitis and pulmonary hypertension). RA-ILD occurs in 10%–30% of patients, and is the only complication of RA increasing in prevalence [33,82]. Prognosis is poor, with a mean survival from diagnosis of 3–8 years, accounting for approximately 6% of all RA deaths [82,83,84]. Established risk factors for RA-ILD include male sex, older age and smoking, with high titre rheumatoid factor (RF) and anti-citrullinated cyclic peptide antibodies (anti-CCP) as predictive and prognostic markers [33,85,86].

For the diagnosis of RA, anti-CCP has higher specificity compared with RF (95–99% vs. 80–86% respectively) and similar sensitivity (50–88%) [87,88,89]. It remains unclear whether combining RF and anti-CCP improves sensitivity [90]. There is increasing evidence that anti-CCP antibodies are pathogenic, whereby environmental events such as smoking trigger the generation of citrullinated proteins and autoantigens in the lungs of susceptible individuals, and the development of autoantibodies then induces systemic inflammation and autoimmunity [91,92].

Anti-CCP antibodies are highly specific for RA and predictive for erosive joint disease. Anti-CCP-positive-RA forms a unique patient subset, with a distinct natural history from anti-CCP-negative-RA [93]. Overall, studies demonstrate increased risk of RA-ILD in patients demonstrating anti-CCP or high-titre RF [86,94,95,96]. Occasionally anti-CCP antibodies are detected in other CTDs (such as SLE, SSc and Sjögren’s syndrome), but in one study of RA patients, the presence of concurrent CTDs did not affect the association with RA-ILD [96]. The implications of the presence of anti-CCP antibodies and ILD in the absence of a diagnosis of RA remains unknown and will be discussed later [94,97,98].

Small cohort studies have demonstrated a possible correlation between anti-CCP titres and the severity of RA-ILD (measured by HRCT fibrosis score) [93,95]. However, data regarding longitudinal disease behaviour is generally lacking and currently, no clinical variable (including demographic, radiological, serological and physiological parameters), or a clinical model combining these can accurately predict mortality in RA-ILD [84].

### 3.3. Myositis Autoantibodies (Including tRNA Synthetase Antibodies)

Idiopathic inflammatory myopathies (IIM) are a group of systemic autoimmune conditions including polymyositis (PM) and dermatomyositis (DM). Myositis autoantibodies are present in up to 40% of patients with myositis (either PM or DM) and are associated with an increased risk of ILD [35,99]. The major myositis autoantibodies and their association with myositis-associated ILD are shown in Table 3. ILD occurs in 30–50% of myositis patients and is a major determinant of disease prognosis and survival [34,35].

#### 3.3.1. Anti t-RNA Synthetase Antibodies

The most common myositis antibodies are anti-tRNA synthetase antibodies, present in 25–35% of all IIM patients [35,99,100]. ANA may be present in <50% of patients with anti-tRNA synthetase antibodies, thus further evaluation for specific myositis antibodies should be undertaken in the appropriate clinical circumstances [101,102].

Of the eight identified anti-synthetase antibodies, anti-Jo-1 is the most common, detectable in 20–30% with PM and 2–10% with DM [35,103,104,105]. Anti-PL-7 and PL-12 antibodies are found in 3–4% of patients with myositis, and the remaining anti-synthetases (anti-OJ, EJ, KS, Ha and Zo) found in <2% [104,106].

The presence of anti-synthetase antibodies is thought to characterise a unique phenotype: the “anti-synthetase syndrome”, which carries substantial risk of developing ILD. Diagnosis of anti-synthetase syndrome is very challenging due to the wide variability in the degree and timing of other clinical features that may include myositis, arthritis, Raynaud’s phenomenon, mechanic’s hands, skin rashes, sicca syndrome and fever [100]. Furthermore, in a significant proportion, ILD may be the dominant symptom with no muscle-related or dermatologic disease at presentation [34,100,107]. There remains no standardised criteria for anti-synthetase syndrome, and Connors et al. recently proposed the presence of relevant autoantibodies and ILD as the sole criteria for diagnosis, but this proposal still requires validation (outlined in Table 4) [35]. 

It remains unclear how to interpret the presence of anti-synthetase antibodies in the absence of any other features of myositis or CTD, whether the different anti-synthetase antibodies represent distinct phenotypes (e.g., anti-Jo-1 versus anti-PL-7/PL-12), and what the implications for management are. 

#### 3.3.2. Anti CADM140/MDA5 Antibodies

The anti-CADM140 antibody, also known as anti-MDA5 targeting melanoma differentiation associated gene 5, is found in 20–30% of patients with DM and characterises the clinical subset “clinically amyopathic DM” (CADM). Up to 50% of patients with detectable anti-CADM antibodies develop rapidly progressive-ILD, with a 40–55% 6-month survival rate and poor response to immunosuppressive therapy [43,108,109,110]. Studies have suggested that anti-CADM titres may help monitor disease activity and treatment response, but similar to the difficulties faced with the anti-synthetase syndrome, there is no clear definition for CADM and research on a uniform cohort is urgently required [108,111].

#### 3.3.3. Anti-Mi2 Antibodies

The anti-Mi2 antibody is found in 10–30% of patients with DM (specificity 98–100%, sensitivity 18%), and is strongly associated with skin manifestations and a low risk of pulmonary disease [105]. However, the literature is very limited.

#### 3.3.4. Anti-SRP Antibody

Anti-signal recognition particle (SRP) antibodies are specific for myositis, found in 4–8% of PM patients and associated with severe necrotising myopathy and poor response to treatment, but no association with ILD has been described to date [105,107].

### 3.4. Anti-SSA/Ro60, Anti-Ro52 and Anti SSB/La Antibodies

Anti-SSA/Ro and anti-SSB/La antibodies target three different proteins (52 kDa Ro, 60 kDa Ro and La). Anti-SSA/Ro antibodies are reported in many CTDs including Sjögren’s syndrome (SS), SSc, SLE, Sjögren’s/SLE overlap, subacute cutaneous lupus erythematosus, RA and DM [55,112]. Anti-SSA/Ro antibodies are detectable in up to 15%–20% of patients with SSc and may confer increased risk of SSc-ILD [55]. The *German Network for Systemic Sclerosis* (reporting on 863 SSc patients) and the *Canadian Scleroderma Research Group* (963 patients) described an odds ratio for SSc-ILD of 2.20 and 2.86 respectively with anti-SSA/Ro60 positivity [113,114]. In SLE, anti-SSA/Ro antibodies have been associated with later onset disease, and an increased prevalence of ILD and neurologic features, although data is very limited [115,116].

Anti-SSB/La antibodies are largely associated with SS, although its presence alone without detectable anti-SSA/Ro is no longer considered a criterion item for diagnosis of SS [112,117]. Primary SS is a systemic autoimmune disease affecting exocrine glands, resulting in xerostomia/dry-mouth and xerophthalmia/dry-eyes (“sicca syndrome”), with variable extraglandular and lung involvement. Population-based estimates of SS-associated ILD range from 3% to 11% and is associated with worse survival [36,118]. In a recent multi-centre study of 263 French patients with SS, there was a non-significant trend towards more frequent ANA-positivity in patients with ILD, but no association with anti-SSA/Ro or anti-SSB/La antibodies [36].

Anti-SSA/Ro and anti-SSB/La antibodies have also been described in inflammatory myopathies, particularly the anti-synthetase syndrome and myositis overlap syndromes with SLE and SS [104]. Small cohort studies have demonstrated more severe ILD (defined as greater extent of fibrosis on HRCT and impairment of pulmonary function measures), and greater resistance to immunosuppressive therapy in anti-Jo-1 positive myositis patients with concomitant anti-SSA/Ro antibodies compared with anti-SSA/Ro negative patients [119,120]. The impact on survival and long-term outcomes remains unclear [119,120].

### 3.5. Anti-dsDNA and Anti-Sm Antibodies

Antibodies to double-stranded DNA (anti-dsDNA) and anti-Smith (Sm) antibodies are both highly specific for the diagnosis of SLE [39]. Chronic diffuse ILD occurs in 3–8% of SLE patients, is more common in older patients, males and in late-onset SLE, with a more indolent disease course compared with idiopathic ILD [121,122,123]. Onset can be insidious or following acute lupus pneumonitis [123]. Anti-dsDNA and anti-Sm antibodies have demonstrated increased risk of renal and cutaneous involvement in SLE, but no correlation with SLE-ILD has been described in large observational European and Chinese cohorts [124,125,126,127].

## 4. Autoantibodies and Interstitial Pneumonia with Autoimmune Features

Diagnosing or excluding an underlying CTD is a key component in the assessment of patients with ILD. Yet a proportion of individuals with ILD will have autoimmune features, but do not fulfil complete diagnostic criteria for a defined CTD. Nomenclature previously proposed for such patients has included “lung dominant CTD-ILD”, “autoimmune-featured ILD” (AIF-ILD) and “undifferentiated CTD-associated ILD” (UCTD-ILD) [9,128,129]. Without uniform disease criteria, systematic characterisation of a comparable cohort has hitherto not been possible. Assayag et al. applied four previously published criteria (Kinder, Vij, Corte, and Fischer [9,128,129,130]) for the general entity of “ILD with features of autoimmunity” to 119 ILD patients, and found that only 18% met all four criteria [131].

In 2015, an European Respiratory Society/American Thoracic Society (ERS/ATS) taskforce proposed the research entity “interstitial pneumonia with autoimmune features” (IPAF) to allow characterisation of a uniform cohort with the aim of developing a consensus classification criteria for such individuals [10]. The IPAF criteria is organised around three central domains: clinical, serological and morphological, with the full criteria shown in Table 5.

The serological domain includes high ANA titre ≥ 1:320 and RF level > 2 times the upper limit of normal, based on previous studies that demonstrate ANA and RF at these levels is more commonly associated with UCTD-ILD and AIF-ILD when compared with idiopathic ILD, where the ANA titre is more commonly ≤1:80 [9,128,129,132]. With the aim of finding a balance between being too “broad” or “narrow”, less specific serologic markers such as erythrocyte sedimentation rate (ESR), C-reactive protein (CRP) and creatine phosphokinase (CK) were not included in the IPAF criteria [10]. 

Early retrospective studies describing the clinical phenotype and natural history of patients with IPAF have demonstrated that in the majority of patients, the presence of autoantibodies play a major role pointing towards an underlying autoimmune process. Chartrand et al. demonstrated that 91% of their re-classified IPAF cohort had at least one serological feature, with ANA ≥1:320 being the most common serologic finding, followed by anti-SSA and RF [133]. This echoes a prior study of 144 ILD patients by Oldham et al., in which at least one serological feature was demonstrable in 91.7% of patients who met IPAF criteria [134].

However, disease behaviour and outcomes in IPAF and the impact of specific autoantibody positivity in ILD without a CTD remains unclear. Vij et al. demonstrated worse survival in patients with AIF-ILD compared with CTD-ILD, and improved survival in AIF-ILD patients with an ANA titre ≥1:1280 [129]. Oldham et al. also found that survival was markedly worse in their modified IPAF cohort compared with CTD-ILD patients, but this was partly driven by the underlying radiographic and/or histological pattern and not associated with ANA positivity [134].

Yamakawa et al. compared the baseline and survival characteristics of patients with SSc-ILD and SSc-antibody-positive-ILD (ScAb-ILD), who had detectable anti-centromere, anti-Scl70 and/or anti-U1 RNP antibodies but did not fulfil diagnostic criteria for SSc [135]. Most of the subjects with ScAb-ILD matched diagnostic criteria for IPAF by fulfilling serologic and morphologic criteria. Patients with SSc-ILD were predominantly females and non-smokers, with a radiological pattern of NSIP on HRCT [135]. In contrast, half of the ScAb-ILD patients were male and current/ex-smokers, with more predominant honeycombing on HRCT and less severe vascular thickening on pathological analysis [135]. The ScAb-ILD patients demonstrated significantly worse survival than those with SSc-ILD (cumulative 5-year mortality 10.9% versus 35.9% respectively, *p* = 0.011), and authors hypothesised that SSc-ILD and ScAb-ILD may represent distinct entities [135].

In contrast, a small series of ILD patients with anti-tRNA synthetase antibodies with and without features of PM/DM were examined by Takato et al. and the two groups demonstrated no significant difference in radiological, cytological and physiological manifestations of pulmonary disease or response to immunosuppressive therapy [136]. Fischer et al. described a cohort of patients with lung disease and anti-CCP antibody positivity but without evidence of RA or another CTD [97]. Individuals had similar pulmonary phenotypic features to patients with established RA, and they discussed whether such individuals represent a “pre-RA” state requiring more rigorous monitoring for the development of synovitis [97].

The presence of a specific autoantibody alone in an individual with ILD may be the only indication of autoimmunity, and should all other aetiologies be excluded, the diagnosis will remain idiopathic ILD. In small series, no survival difference has been demonstrated comparing IPF patients with and without autoantibodies, and whether these groups have differing clinical phenotypes remains unclear [30,132].

These studies demonstrate the urgent need for prospective, multi-centre studies to validated the proposed IPAF criteria, and determine the natural history and clinical implications of IPAF and autoantibody positivity in individuals with parenchymal lung disease but without a CTD, and how this may impact prognosis and management compared with IIP and CTD-ILD.

## 5. Discussion and Conclusions

The diagnosis of CTD-ILD is challenging due to the wide spectrum of disease entities encompassed and considerable heterogeneity in disease phenotypes between populations. Immunologic tests serve an important role detecting potentially unrecognised CTD in ILD patients, especially when other dermatologic, arthritic or myopathic features are subtle or absent, but there is no consensus about which serological tests to obtain at first encounter. Furthermore, which tests should be repeated during follow-up to detect the significant proportion of patients with CTD-ILD who demonstrate seroconversion or whether serial measures play a role monitoring disease progression also remains unknown.

Overall, it remains unclear if autoantibodies can predict outcomes or survival in CTD-ILD. Autoantibodies such as anti-tRNA synthetase and anti-CADM/MDA5 antibodies potentially play a vital role identifying disease-specific phenotypes and predicting risk of more progressive ILD requiring early aggressive treatment, yet their low frequency and need for specialised assays currently limits large prospective clinical research and widespread use.

There is also a lack of standardisation of immunological testing techniques between laboratories, compounded by non-uniform definitions for many CTD entities, such as the idiopathic inflammatory myopathies and anti-synthetase syndrome. This results in significant variability in reported test sensitivities and specificities and an inability to accurately pool results. Thus, despite increasing awareness that distinguishing CTD-ILD and IIP has vital implications for management and prognosis, which autoantibody tests to perform and the subsequent management of ILD patients based on these results is often left to the individual health care provider. 

The implications of detecting autoantibodies in patients with no or incomplete CTD/autoimmune features on disease behaviour and patient management remains unclear and the need to standardise our approach to patients undergoing assessment for ILD is highlighted by the proposed ERS/ATS criteria for IPAF. Whilst largely a research classification with unknown clinical implications currently, this represents an important first step towards building a minimum dataset through research on a uniform cohort of this poorly understood group of patients.

As laboratory techniques become more sensitive, detection of autoantibodies is likely to increase and thus it is with urgency that robust, prospective research to validate preliminary findings and answer these questions occurs. Autoantibodies have the potential to improve accuracy of diagnosis and in the future potentially individualise treatment strategies. However, it remains imperative that immunologic tests are requested and interpreted within the patient’s overall clinical context and in conjunction with a detailed rheumatologic history and examination. Optimal management of the patients with CTD-ILD demands effective multidisciplinary collaboration between respiratory physicians, rheumatologists, immunologists, radiologists and pathologists to yield a more complete diagnosis.

## Figures and Tables

**Table 1:** Prevalence and radiologic/histologic pattern for interstitial lung disease (ILD) in major connective tissue diseases.

CTD	Prevalence of ILD	Radiological/Histopathological Pattern
SSc	40–75% with clinically significant disease (at least moderate impairment on pulmonary function) [11,31,32] Up to 70% with detectable interstitial changes on HRCT [31]	Most common: NSIP Other: UIP
RA	Detectable on HRCT: 30–60% Clinically evident 10–30% [33]	Most common: UIP Other: NSIP, OP, LIP
IIM	30–50% [34,35]	Most common: NSIP Other: UIP, OP, DAD
SLE	3–11% chronic diffuse interstitial disease [36] Up to 30% with detectable interstitial changes on HRCT Need to distinguish from acute pneumonitis (1–10%) and alveolar haemorrhage (rare)	Most common: NSIP Other: LIP, OP, UIP
SS	10–30% [31] Need to exclude pulmonary lymphoma	Most common: NSIP Other: LIP, OP, UIP
MCTD	20–85% [31]	Common: NSIP Other: UIP

Note: CTD, connective tissue disease; HRCT, high resolution computed tomography; SSc, systemic sclerosis; RA, rheumatoid arthritis; IIM, idiopathic inflammatory myopathy; SLE, systemic lupus erythematosus; SS, Sjögren’s syndrome; MCTD, mixed connective tissue disease; NSIP, non-specific interstitial pneumonia; UIP, usual interstitial pneumonia; OP, organising pneumonia; LIP, lymphocytic interstitial pneumonia; DAD, diffuse alveolar damage.

**Table 2:** Major autoantibodies associated with CTDs.

Autoantibody	Associated CTD(s)
Antinuclear antibody (ANA; ≥1:320)	SSc, SLE, Sjögren’s, PM/DM
*Systemic sclerosis associated*	
Anti-topoisomerase (ATA/anti-Scl70)	SSc (diffuse)
Anti-centromere	SSc (limited)
Anti-RNA polymerase (RNA-pol)	SSc
Anti-Th/To	SSc
Anti-PM/Scl-75/100	SSc-myositis overlap, SLE, Sjögren’s
Anti-U3 ribonucleoprotein (anti-U3 RNP)	SSc
Anti-U1 ribonucleoprotein (anti-RNP or anti-U1 RNP)	SSc-overlap, MCTD
Anti-U11/U12 ribonucleoprotein (anti-U11/U12 RNP)	SSc
*Rheumatoid arthritis associated*	
Rheumatoid factor (≥60 IU/mL)	RA, Sjögren’s, SLE
Anti-cyclic citrullinated peptide (anti CCP)	RA
*Myositis associated*	
Anti-synthetase (Jo-1, PL-7, PL-12, EJ, OJ, KS)	PM/DM (anti-synthetase syndrome)
Anti-Mi2	PM/DM
Anti-CADM140 (anti-MDA5)	Clinically amyopathic DM
*Overlap syndromes*	
Anti-Ku	SSc, SSc-PM overlap, SLE, myositis,
Anti SS-A/Ro, anti SS-B/La	Sjögren’s, SLE, Sjögren’s/SLE overlap, SSc, RA, DM
*Systemic lupus erythematosus associated*	
Anti ds-DNA	SLE
Anti-Smith	SLE

Note: SSc, systemic sclerosis; SLE, systemic lupus erythematosus; PM/DM, polymyositis/dermatomyositis; MCTD, mixed connective tissue disease; RA, rheumatoid arthritis.

**Table 3:** Myositis autoantibodies associated with ILD.

Autoantibody	Clinical Associations
*Myositis specific autoantibodies*	
Anti- tRNA synthetases (Jo-1, PL-7, PL-12, EJ, OJ, KS, Ha, Zo)	PM, DM, anti-synthetase syndrome
Anti-Mi-2	“Classic DM”; lung-sparing
Anti- CADM140 (MDA5)	Clinically amyopathic DM ILD; poor prognosis
Anti-SRP	Severe necrotising myopathy; association with ILD not described
*Myositis associated antibodies*	
Anti-Ro/SSA	PM/Sjögren’s overlap; severe ILD
Anti-PM/Scl	PM/Scleroderma overlap; severe ILD
Anti-Ku	PM/Scleroderma overlap; severe ILD
Anti-U1RNP	PM/SLE overlap; ILD

Note: tRNAs: transfer RNAs, PM: Polymyositis, DM: Dermatomyositis, MDA-5: Melanoma differentiation-associated protein 5. Adapted from Ghirardello A et al., Myositis autoantibodies and clinical phenotypes, *Autoimmunity Highlights* **2014**, *5*, 69–75, with permission of Springer.

**Table 4:** Proposed criteria for anti-synthetase syndrome.

Patient must have: Positive serologic testing for an anti-tRNA synthetase autoantibody Plus one or more of the following conditions: Evidence of myositis by Bohan and Peter criteria Evidence of ILD by ATS criteria Evidence of arthritis by clinical examination, radiographic findings, or patient self-report Unexplained, persistent fever Raynaud phenomenon Mechanic’s hands

Note: ATS American Thoracic Society. Reprinted from CHEST, vol 138, Connors et al., Interstitial lung disease associated with the idiopathic inflammatory myopathies: what progress has been made in the past 35 years? p. 1467, Copyright (2010), with permission from Elsevier.

**Table 5:** Proposed criteria for interstitial pneumonia with autoimmune features (IPAF).

Presence of an interstitial pneumonia by HRCT or surgical lung biopsy Exclusion of alternative aetiologies Does not meet criteria for a defined CTD Has at least one feature from at least two of the following domains:		
**A. Clinical domain**	**B. Serological Domain**	**C. Morphological domain**
Distal digital fissuring (i.e., “Mechanic hands”) Distal digital tip ulceration Inflammatory arthritis *or* polyarticular morning joint stiffness ≥60 min Palmar telangiectasia Raynaud’s phenomenon Unexplained digital oedema Unexplained fixed rash on the digital extensor surfaces (Gottron’s sign)	ANA ≥1:320 titre, diffuse, speckled, homogeneous patterns *or* ANA nucleolar pattern (any titre) *or* ANA centromere pattern (any titre) RF ≥2 × ULN Anti-CCP Anti-dsDNA Anti-Ro (SS-A) Anti-La (SS-B) Anti-ribonucleoprotein Anti-SmithAnti-topoisomerase (Scl-70) Anti-tRNA synthetase (e.g., Jo-1, PL-7, PL-12, others are: EJ, OJ, KS, Zo, Ha) Anti-PM/Scl Anti-CADM140 (anti-MDA5)	**1. Suggestive radiology patterns by HRCT** NSIP OP NSIP with OP overlap LIP **2. Histopathology patterns or features by surgical lung biopsy:** NSIP OP NSIP with OP overlap LIP Interstitial lymphoid aggregates with germinal centres Diffuse lymphoplasmacytic infiltration (with or without lymphoid follicles) **3. Multi-compartment involvement (in addition to IP):** Pleural effusion or thickening (not otherwise explained) Pericardial effusion or thickening (not otherwise explained) Small airways disease (by PFTs, imaging or pathology) Pulmonary vasculopathy

Note: Reproduced from Fischer, A.; Antoniou, K.M.; Brown, K.K. An official European Respiratory Society/American Thoracic Society research statement: Interstitial pneumonia with autoimmune features. *Eur. Respir. J.* **2015**, *46*, 976–987.
